# Bats—The Magnificent Virus Player: SARS, MERS, COVID-19 and Beyond

**DOI:** 10.3390/v15122342

**Published:** 2023-11-29

**Authors:** Kenneth S. M. Li, Susanna K. P. Lau, Patrick C. Y. Woo

**Affiliations:** 1 Department of Microbiology, School of Clinical Medicine, Li Ka Shing Faculty of Medicine, The University of Hong Kong, Hong Kong 999077, China; kenn105@hku.hk (K.S.M.L.); skplau@hku.hk (S.K.P.L.); 2 Doctoral Program in Translational Medicine and Department of Life Sciences, National Chung Hsing University, Taichung 402, Taiwan; 3 The iEGG and Animal Biotechnology Research Center, National Chung Hsing University, Taichung 402, Taiwan

Irrespective of whether COVID-19 originated from a natural or a genetically engineered virus, the ultimate source of Severe Acute Respiratory Syndrome coronavirus 2 (SARS-CoV-2) is bats. Since the emergence of SARS in 2003 and the discovery of SARS-related coronavirus from Chinese horseshoe bats (*Rhinolophus sinicus*) [1] and subsequently other horseshoe bat species, including greater horseshoe bats (*Rhinolophus ferrumequinum*), big-eared horseshoe bats (*Rhinolophus macrotis*), least horseshoe bats (*Rhinolophus pusillus*), intermediate horseshoe bats (*Rhinolophus affinis*) and Blasius’s horseshoe bats (*Rhinolophus blasii*) [2,3,4,5], studies on the relationships between this group of animals and various virus families have surged in an exponential manner. In regard to studies between bats and coronaviruses, cell lines from a variety of bat species, such as lesser bamboo bats (*Tylonycteris pachypus*), the hosts of *Tylonycteris* bat coronavirus HKU4; Japanese pipistrelle (*Pipistrellus abramus*), the hosts of *Pipistrellus* bat coronavirus HKU5; Chinese horseshoe bats (*R. sinicus*), the hosts of SARS-related coronavirus and *Rhinolophus* bat coronavirus HKU2; Pomona roundleaf bats (*Hipposideros Pomona*); small bent-winged bats (*Miniopterus pusillus*); and Rickett’s big-footed bats (*Myotis ricketti*), have been developed and used for the study of SARS-related coronavirus (including SARS-CoV and SARS-CoV-2) and MERS coronavirus [6,7]. In addition to the development of bat cell lines, *Tylonycteris* bat coronavirus HKU4, a MERS-related CoV from lesser bamboo bats, has been isolated and could replicate in human colorectal adenocarcinoma (Caco-2) and human hepatocarcinoma (Huh7) cells with cytopathic effects [8]. It has also been shown that similar to MERS coronavirus, *Tylonycteris* bat coronavirus HKU4 was also able to utilize human dipeptidyl-peptidase-4 and dromedary camel dipeptidyl-peptidase-4 as the receptors for cell entry. This represents the first bat coronavirus that has been successfully isolated. 

Apart from coronaviruses, progress in our understanding between bats and other virus families has been made. Lloviu virus, a filovirus which was identified in Schreiber’s bats (*Miniopterus schreibersii*) in Spain in 2002, was successfully isolated from the blood of a Schreiber’s bat in Hungary [9]. The virus was cultured in the kidney cell line SuBK12-08 developed from *Miniopterus* bat, and was shown to be able to infect monkey and human cells [9]. For influenza virus, Giotis et al. demonstrated that the entry of the bat influenza H17N10 virus into mammalian cells was enabled by the major histocompatibility complex class II HLA-DR receptor [10]. In another study, Ciminski et al. showed that bat influenza viruses, such as H18N11, were able to transmit among bats, but were poorly adapted to non-bat species such as mice and ferrets [11]. Moreover, the authors also demonstrated that N11 is most likely the determining factor of influenza virus transmission in bats. For the rabies virus, using hundreds of rabies viruses collected from more than 20 bat species in North America, Streicker et al. were able to reconstruct the evolutionary history of viral establishment in new hosts [12].

In addition to individual virus families, studies demonstrating co-infection of different viruses in bats have been performed. In one study, the authors found a high frequency of virus co-infection in bats from Yunnan province, which is one of the provinces in China with the highest diversity of animals [13]. In another systematic review, Jones et al. reported that viral co-infection in bats is common, although such findings are often ad hoc by-products of viral discovery efforts, instead of results of co-infection studies [14]. Interestingly, it was shown in a recent study using next-generation sequencing that divergent insect- and bat-associated viruses, namely densovirus, nodavirus, jingmenvirus, bastrovirus, dicistrovirus, picornavirus and cyclovirus, were detected in the plasma of two individuals from Africa who were enrolled in a blood-borne surveillance program [15]. Co-infections of bats by two or more viruses of the same family may facilitate virus recombination and reassortment in the corresponding animal. Moreover, co-infections of viruses of different families may rarely lead to a gene encoding a particular protein moving from one virus family to another. The possession of the haemagglutinin esterase gene of influenza C virus in the subgenus *Embecovirus* (lineage A) of *Betacoronavirus* may be a result of such co-infections. 

In addition to the viruses present in bats, advancements in our understanding of bats’ immune systems in relation to viral infections have occurred in recent years. For example, in one study, Ahn et al. showed that the bat apoptosis-associated speck-like protein containing a CARD (ASC2) is highly expressed and is highly potent in inhibiting human and mouse inflammasomes [16]. Transgenic expression of bat ASC2 in mice dampened inflammation induced by multiple viruses, reduced mortality from influenza A virus infection and suppressed SARS-CoV-2 immune-complex-induced inflammasome activation [16]. In another study, Friedrichs et al. utilized single-cell RNA sequencing and immunostaining panels for the characterization of the immune cell landscape in juvenile, subadult and adult Egyptian rousette bats (*Rousettus aegyptiacus*) [17]. The results showed that different immune cell subsets predominated in bats of different ages and provided a comprehensive map of the age-dependent immune landscape of these bats [17].

As with the COVID-19 pandemic, vaccines have been shown to be the most effective method of controlling disease and virus spread. With the previous efforts of hunting coronaviruses hosted in bats, the counterparts of MERS-CoV and SARS-CoV-2 with bat origin were swiftly identified, which aided in the study of these human pandemic CoVs such as receptor usage and vaccine development. While COVID-19 is seemingly “under control”, MERS-CoV is still circulating in the Middle East and will likely cause another outbreak. However, the MERS vaccine is still not available and hence there is an urge for prompt vaccine development. In preparation of future pandemics, whether caused by coronaviruses or other viruses, it is necessary to have regular viral surveillance in bat populations since bats host many diverse viruses and possess a number of unique characteristics. First, there are more than 1400 bat species. In fact, among all mammals, bats have the second largest number of species, only second to rodents. Such a large number of bat species implies that bats possess a large variety of cells and cellular receptors, which are able to host a high diversity of viruses. For example, in coronaviruses, different bat species are hosts of different coronaviruses, with few exceptions [18]. Second, the ability of bats to fly has enabled them to disseminate viruses in their bodies. These two characteristics are similar to those of birds, which also have a large number of species and the capability of flying. Third, the fact that hundreds of bats have the habit of living together has facilitated the transmission of viruses among bats. Fourth, one bat species could be the host of more than one virus of the same family; for example, Chinese horseshoe bats are the host of SARS-related coronavirus and *Rhinolophus* bat coronavirus HKU2 [19]. The presence of multiple viruses in the same bat can facilitate recombination among different viral species in the same family. All of these properties of bats will continue to place this group of animals at the forefront of research.
